# Enhancing Familiarity and Utility: A Pre–Post Survey Study on Mental Practice Workshop Outcomes

**DOI:** 10.1177/23821205241299583

**Published:** 2024-12-12

**Authors:** Carlos Hölzing, Benjamin Gordon, Patrick Meybohm, Nadia Spitznagel, Stephen Hearns, Oliver Happel

**Affiliations:** 1 Department of Anaesthesiology, Intensive Care, Emergency and Pain Medicine, 27207 University Hospital Würzburg, Würzburg, Germany; 2 Klinik für Anästhesie und Intensivmedizin, Palliativ- und Schmerzmedizin, 9210 Klinikum Ingolstadt, Ingolstadt, Germany; 3 ScotSTARHangar B, Emergency Medical Retrieval Service, Paisley, UK

**Keywords:** cognition, medical education, clinical competence, psychological stress

## Abstract

**OBJECTIVES:**

Mental practice, a cognitive technique for practicing skills without physical movement, holds promise for enhancing clinical outcomes in emergency medicine. This study investigates its recognition and the impact of a basic workshop on emergency physicians’ attitudes toward mental practice.

**METHODS:**

This pre–post survey study involved 20 medical professionals who participated in a 2-day workshop. Assessments were conducted before and after the intervention using a structured questionnaire.

**RESULTS:**

Initial findings revealed that 65% of participants were aware of mental practice, and only 10% utilized it in clinical settings. Postworkshop, familiarity, and perceived helpfulness significantly increased from 2.2 to 4.1 and 3.9 to 4.7, respectively, with marked improvements in confidence and the intention to apply these techniques clinically by 100% of the participants.

**CONCLUSION:**

The results suggest that brief educational interventions can substantially influence medical professionals’ engagement with mental practice, advocating its inclusion in medical training curricula to enhance procedural skills and patient care.

## Background

Mental stimulation, also referred to as mental practice, is a technique that is utilized in professional sports regularly and has gained significant attention in the field of emergency medicine lately. It involves the cognitive practice of a skill without physical movement, allowing individuals to practice procedures or scenarios mentally through visualization. Those working in the field of emergency medicine are frequently required to make crucial decisions under considerable time pressure, a situation that can give rise to considerable stress and a state of cognitive overload.^1^ These stressful situations, such as an unwell young pediatric patient and an unwell pregnant patient require not only technical proficiency but also the ability to remain calm and focused.^2^

The cognitive and neural mechanisms underlying mental practice involve the activation of neural pathways associated with the actual performance of tasks.^3^ The dorsal premotor cortex plays a crucial role in this process, as evidenced by behavioral and imaging studies which indicate that mental practice activates similar neural pathways as those engaged during actual motor performance.^3^ This is particularly evident in the activation of motor circuits, which occurs not only during the observation of actions but also when individuals are provided with information that specifies a particular action.^3^ This neural overlap suggests that mental practice not only aids in skill acquisition but also reinforces motor memory, making it a valuable tool for learning and performance optimization.^4^ Studies have shown that mental practice can enhance the acquisition of technical and procedural skills.^5^ Moreover, mental practice has been linked to improved decision-making processes among novice emergency physicians, indicating its potential to enhance clinical outcomes.^6^ As an experiment, mental practice has been incorporated into surgical training to improve performance and reduce the stress levels of the practitioners.^7^

The aim of our study was to evaluate whether a basic workshop could influence emergency physicians’ attitudes toward mental practice.

## Methods

This study employed a pre–post questionnaire design to evaluate the impact of a 2-day workshop on performance in stressful situations. The workshop included a 45-min session on mental practice, conducted among 20 medical professionals (experienced specialists and young residents with at least 2 years of experience). Data collection occurred before (March 8, 2024) and after (March 9, 2024) the workshop.

The training program commenced with a comprehensive theoretical introduction to the significance and methodology of mental practice. In order to facilitate the transfer of knowledge, instructional materials were distributed and a lecture with an infographic was delivered (Supplement no. 1). Subsequently, the practical application phase commenced. During the practical phase, participants were instructed to wear hearing protection and blindfolds in order to minimize external sensory stimuli and enhance focus on mental exercise. The session commenced with a breathing exercise, the objective of which was to clear cognitive resources and reduce distracting thoughts, thus preparing the participants for the subsequent mental practice activities. Subsequently, the participants were guided through a complex emergency scenario in a step-by-step manner. The participants were instructed to mentally simulate each action and communication in detail, with particular emphasis on visualizing the auditory, olfactory, and environmental elements, such as the soundscape, smells, and overall atmospheric conditions of the simulated situation. Following the completion of the simulated emergency situation, the participants were given a brief period of rest, after which they engaged in a feedback session. During this session, they were encouraged to reflect on the process, discuss their experiences, and assess the impact of mental practice on their cognitive and emotional preparedness.

Written informed consent was obtained from all participants before the questionnaires were administered. The internally created questionnaire was pretested with 4 project team members. Questionnaires assessed participants’ confidence in mental practice and the applicability of mental practice techniques (Supplement no. 2).

### Statistical analysis

Data analysis using IBM^®^ SPSS involved descriptive statistics and the Mann–Whitney *U* test for quantitative data. Ethics approval for the study was not required as the data collected was completely anonymous and only involved interviewing employees anonymously as part of quality management. This is in accordance with Section 15 Research of the Professional Code of Conduct for Doctors in Bavaria.

## Results

**Figure 1.**
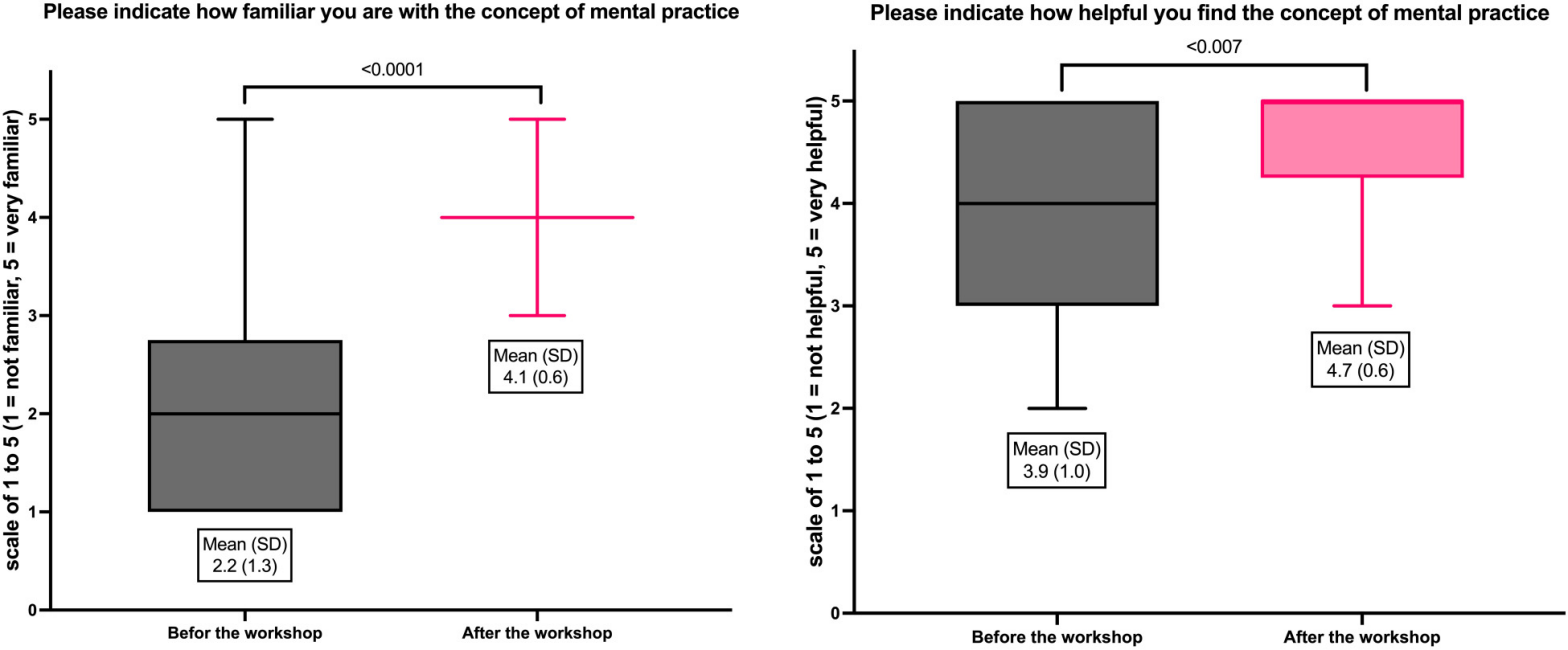
Changing attitudes toward mental practice.

Before the training, 65% (*n* = 13) of participants were aware of mental simulation/practice; however, only 10% (*n* = 2) frequently used it in clinical practice. Initially, their familiarity was rated at 2.2 ± 1.3 (scale 1–5, median = 2), and perceived helpfulness scored 3.9 ± 1.0 (median = 4). Posttraining, familiarity increased to 4.1 ± 0.6 (median = 4) (*p* < 0.0001, *U* = 351.50), and the concept's helpfulness to 4.7 ± 0.6 (median = 5) (*p* = 0.007, *U* = 292.00) (see Figure 1). The training's effectiveness was rated at 4.3 ± 0.7 (median = 4). Postworkshop, 70% (*n* = 14) reported significant knowledge and skill improvements, and 95% (*n* = 19) felt more confident in using the technique clinically. Notably, every participant expressed the intention to adopt the technique.

## Discussion

The study highlights a significant gap between the awareness of mental practice among emergency physicians and its practical application. While mental practice is recognized in other high-stakes fields, such as sports, it remains underutilized in emergency medicine despite its potential benefits. Initial data showed that while the majority of participants were aware of mental practice, only very few actively used it in their clinical practice. The intervention, a concise workshop, remarkably increased both familiarity with (from a mean score of 2.2 to 4.1) and perceived helpfulness of (from 3.9 to 4.7) mental practice techniques. These substantial improvements, evidenced by significant statistical changes (*p* < 0.0001 for familiarity; *p* = 0.007 for helpfulness), suggest the efficacy of brief educational programs in modifying attitudes toward mental practice and hopefully even clinical practice behaviors. Postworkshop outcomes demonstrated significant knowledge and skill enhancements, with 95% of participants reporting increased confidence in applying mental practice clinically. Moreover, all participants (100%) expressed their intention to adopt mental practice in the future. This unanimous intention indicates that with proper training and exposure, mental practice could become a standard practice in emergency medicine, similar to its use in other fields. By enhancing familiarity and confidence in mental practice, this study suggests that such educational interventions may be effective tools in helping medical professionals manage stress and improve performance in high-pressure environments. These findings advocate for the integration of mental practice techniques into medical training curricula, considering their proven benefits in improving procedural skills, decision-making, and stress management in medical practice.^8,9^

This study is limited by its small sample size, potentially impacting the generalizability of the results. Additionally, the short-term design and reliance on self-reported data may not fully capture long-term retention or the objective effectiveness of mental practice techniques. Future studies should consider larger, diverse cohorts, longitudinal assessments, and objective performance metrics to enhance validity and applicability.

## Conclusion

This study demonstrates the effectiveness of a short educational workshop in enhancing familiarity and perceived utility of mental practice for acute care physicians. After the workshop, significant increases in confidence were observed, and all participants expressed an intention to use mental practice clinically. These findings support the integration of mental practice into medical education to improve clinical skills and patient care. However, it is important to note that these findings may be limited by a small sample size and reliance on self-reported data. Therefore, further comprehensive research is needed to confirm these results and explore any potential long-term effects.

## Supplemental Material

sj-pdf-1-mde-10.1177_23821205241299583 - Supplemental material for Enhancing Familiarity and Utility: A Pre–Post Survey Study on Mental Practice Workshop Outcomes Supplemental material, sj-pdf-1-mde-10.1177_23821205241299583 for Enhancing Familiarity and Utility: A Pre–Post Survey Study on Mental Practice Workshop Outcomes by Carlos Hölzing, Benjamin Gordon, Patrick Meybohm, Nadia Spitznagel, Stephen Hearns and Oliver Happel in Journal of Medical Education and Curricular Development

sj-pdf-2-mde-10.1177_23821205241299583 - Supplemental material for Enhancing Familiarity and Utility: A Pre–Post Survey Study on Mental Practice Workshop Outcomes Supplemental material, sj-pdf-2-mde-10.1177_23821205241299583 for Enhancing Familiarity and Utility: A Pre–Post Survey Study on Mental Practice Workshop Outcomes by Carlos Hölzing, Benjamin Gordon, Patrick Meybohm, Nadia Spitznagel, Stephen Hearns and Oliver Happel in Journal of Medical Education and Curricular Development
