# Supplementary material for: Enhancing Familiarity and Utility: A Pre–Post Survey Study on Mental Practice Workshop Outcomes
Source: J Med Educ Curric Dev. 2024 Dec 12;11:23821205241299583. doi: 10.1177/23821205241299583 (PMC11635951; doi:10.1177/23821205241299583)
Supplement: sj-pdf-1-mde-10.1177_23821205241299583 - Supplemental material for Enhancing Familiarity and Utility: A Pre–Post Survey Study on Mental Practice Workshop Outcomes [file sj-pdf-1-mde-10.1177_23821205241299583.pdf]

MENTAL REHEARSAL

WHAT'S IT GOOD FOR?

Personal preparation for predictable emergency situations involving practical task execution.

Also a useful tool for reflection on personal performance after a high pressure event

HOW DOES IT HELP?

Speeds up our decision to act.

Improves speed, safety & accuracy of task execution

Helps create automatic, intuitive cognition.

SET THE SCENE

Immersion in the process is important.

Quiet room.

Eyes closed

HOW TO REHEARSE

Visualise the situation in real time, step by step.

Think about what you will see, what you will hear and what your hands will feel.

REHEARSE POSITIVELY

Visualise yourself carrying out the task with competence and confidence.

Imagine yourself enjoying the experience.

TEAMWORK

Consider how you will interact with the rest of the team under pressure.

How will you manage your posture, facial expression and tone of voice?

What will others say or do and how will you react?

VARIABLES

Visualise how different scenarios may evolve.

What will your actions be if x occurs or y happens?

REFLECTION

If we would like to reflect on our performance following a pressure event we can use mental rehearsal.

Go through the event in real time, recalling your actions & the actions of others.

Consider the stimuli for your actions

CoreCognition

PERFORMANCE UNDER PRESSURE
