# Supplementary material for: Enhancing Familiarity and Utility: A Pre–Post Survey Study on Mental Practice Workshop Outcomes
Source: J Med Educ Curric Dev. 2024 Dec 12;11:23821205241299583. doi: 10.1177/23821205241299583 (PMC11635951; doi:10.1177/23821205241299583)
Supplement: sj-pdf-2-mde-10.1177_23821205241299583 - Supplemental material for Enhancing Familiarity and Utility: A Pre–Post Survey Study on Mental Practice Workshop Outcomes [file sj-pdf-2-mde-10.1177_23821205241299583.pdf]

1. Welcome

Dear participant,

As part of this workshop, we would like to capture your experiences and opinions through this questionnaire. Please fill out the first section (A) before the workshop and the second section (B) after the workshop. We assure you that all your information will be treated confidentially and evaluated anonymously. Participation in this survey is voluntary.

Thank you for your participation!

2. (A) Before the Workshop - Special Part

2.1 Before this training event, have you heard of mental simulation/mental rehearsal?

- ☐ No
- ☐ Yes

2.2 How often have you used mental simulation/mental rehearsal in your clinical practice?

- ☐ Never
- ☐ Rarely
- ☐ Occasionally
- ☐ Frequently
- ☐ Very frequently

2.3 Please indicate how familiar you are with the concept of mental simulation/mental rehearsal on a scale of 1 to 5 (1 = not familiar at all, 5 = very familiar).

- ☐ 1
- ☐ 2
- ☐ 3
- ☐ 4
- ☐ 5

2.4 Please indicate on a scale of 1 to 5 (1 = not helpful at all, 5 = very helpful) how helpful you find the concept of mental simulation/mental rehearsal.

- ☐ 1
- ☐ 2
- ☐ 3
- ☐ 4
- ☐ 5

3. (B) After the Workshop - Special Part

3.1 Please rate your overall experience during the training event on mental simulation/mental rehearsal on a scale of 1 to 5 (1 = very bad, 5 = excellent).

- ☐ 1
- ☐ 2
- ☐ 3
- ☐ 4
- ☐ 5

3.2 Please indicate how familiar you are now with the concept of mental simulation/mental rehearsal on a scale of 1 to 5 (1 = not familiar at all, 5 = very familiar).

- ☐ 1
- ☐ 2
- ☐ 3
- ☐ 4
- ☐ 5

3.3 Please indicate on a scale of 1 to 5 (1 = not helpful at all, 5 = very helpful) how helpful you find the concept of mental simulation/mental rehearsal.

- ☐ 1
- ☐ 2
- ☐ 3
- ☐ 4
- ☐ 5

3.4 How much have you improved your knowledge and skills in mental simulation/mental rehearsal through the event?

- ☐ Not at all
- ☐ A little
- ☐ Moderately

- ☐ Strongly
- ☐ Very strongly

3.5 Do you now feel more confident applying mental simulation/mental rehearsal in your clinical practice?

- ☐ No
- ☐ Yes
- ☐ Unsure

3.6 Would you like to use mental simulation/mental rehearsal as a tool?

- ☐ No
- ☐ Yes
- ☐ Maybe
